# Genome-Wide Association Study and Candidate Gene Identification for Resistance to Bacterial Stem and Root Rot in Sweetpotato

**DOI:** 10.3390/biology15080643

**Published:** 2026-04-19

**Authors:** Xiangsheng Lin, Xiawei Ding, Shixu Zhou, Hongda Zou, Zhangying Wang, Xuelian Liang, Xiangbo Zhang, Lifei Huang

**Affiliations:** 1 Guangdong Province Key Laboratory of Crop Genetic Improvement, Crops Research Institute, Guangdong Academy of Agricultural Sciences, Guangzhou 510640, China; lxs19990423@163.com (X.L.); dingxiawei0224@163.com (X.D.); shixuzhou2024@stu.scau.edu.cn (S.Z.); zouhongda@gdaas.cn (H.Z.); wangzhangying@gdaas.cn (Z.W.); 2 College of Agriculture and Biology, Zhongkai University of Agriculture and Engineering, Guangzhou 510225, China; liangxuelian2005@sina.com; 3 College of Plant Protection, South China Agricultural University, Guangzhou 510640, China; 4 College of Agriculture, South China Agricultural University, Guangzhou 510640, China

**Keywords:** sweetpotato, *Dickeya dadantii*, bacterial stem and root rot, GWAS

## Abstract

Bacterial stem and root rot (BSRR), caused by *Dickeya dadantii*, poses a severe threat to global sweetpotato production, yet the genetic architecture underlying resistance remains elusive. To dissect these mechanisms, we conducted a high-resolution genome-wide association study (GWAS) on 135 diverse accessions, integrating two-year field phenotyping with best linear unbiased prediction (BLUP) and 6.8 million single-nucleotide polymorphism (SNP) markers. This approach mapped nine quantitative trait loci (QTLs) exhibiting significant allelic dosage-dependent effects, with the major locus, qBSRR.6.1 was the primary discriminator between resistant and susceptible genotypes. Crucially, transcriptomic profiling within these loci revealed distinct expression patterns: *IbTCP5* and *IbERF003* (located in qBSRR.5.1 and qBSRR.6.2) were highly expressed in the susceptible cultivar ‘Xinxiang’ but suppressed in the resistant ‘Guangshu87’. Furthermore, BSRR challenge identified *IbPUB4*, *IbKCS5*, and *IbLig1* as priority candidate genes involved in defense, with expression patterns suggesting roles in ubiquitin-mediated protein turnover, cuticular wax biosynthesis, and DNA repair, respectively. In stark contrast, *IbPUB25* was constitutively upregulated in ‘Xinxiang’, potentially acting as a negative regulator of immunity via degradation of target proteins. These findings elucidate the polygenic, dosage-sensitive nature of BSRR resistance and prioritize specific targets for future functional characterization. Pyramiding favorable alleles of positive candidates while silencing potential negative regulators like *IbPUB25* offers a promising avenue for developing durable, high-resistance sweetpotato varieties.

## Simple Summary

Sweetpotato is a vital food source globally, yet it faces a severe threat from bacterial stem and root rot, a disease that destroys plant stems and roots, causing massive harvest losses. The genes involved in resistance to this disease are not yet identified. To solve this, our team analyzed the complete genetic profiles of 135 diverse sweetpotato varieties grown in fields over two years. We successfully identified nine specific genetic regions associated with resistance. Uniquely, our study reveals that protection functions like a “volume dial”: the more copies of these protective genes a plant inherits, the stronger its defense becomes. This discovery is crucial for sweetpotato, which carries six sets of chromosomes, making its genetics complex. Furthermore, we pinpointed specific genes potentially acting as internal security guards; some appear to be hijacked by bacteria in weak plants, while others remain active to block infection in strong ones. These findings provide a precise genetic roadmap for breeders. By applying this knowledge, scientists can rapidly develop new, highly resistant sweetpotato varieties, ensuring global food security and reducing the reliance on chemical pesticides for farmers worldwide.

## 1. Introduction

*Dickeya dadantii* (syn. *Erwinia chrysanthemi*), ranked among the top ten most notorious phytopathogens globally [1], is the causal agent of bacterial stem and root rot (BSRR) and inflicts devastating damage on sweetpotato production worldwide.

It secretes a broad repertoire of extracellular enzymes—including pectin lyases, cellulases, and proteases—that degrade plant cell wall components, leading to tissue maceration, vascular necrosis, and plant death [2,3]. Severe outbreaks can cause yield losses of 50–100%, particularly in southern China [4], where sweetpotato is a major food and industrial crop [5,6]. Although BSRR was first documented in Georgia, USA, in 1974 [7], progress in elucidating the genetic basis of sweetpotato resistance to BSRR has remained remarkably limited. Past studies have focused on pathogen diagnostics [7], resistance screening [8,9], fungal–bacterial interactions [10], population genomics of *D. dadantii* [11,12], germplasm characterization [13], and the development of standardized resistance assays [14]. However, the molecular genetic mechanisms underlying resistance, particularly the roles of specific resistance gene families, remain poorly understood. Given that CC-NBS-LRR genes represent the largest class of plant resistance (R) genes [15] and WRKY transcription factors are pivotal regulators of defense responses [16], investigating these families is crucial for understanding BSRR resistance.

Yet, studies specifically examining WRKY and CC-NBS-LRR gene families [15,16] or pathogen effectors [17] are limited, and functional validation of resistance genes remains scarce. Durable resistance is rare in cultivated germplasm, and chemical control is environmentally unsustainable and prone to rapid pathogen adaptation [18]. Consequently, deploying natural genetic resistance through breeding constitutes the most durable long-term strategy [19,20]. Successes in other crops, including *Rpi-vnt1.1* in Potato (*Solanum tuberosum*) [21,22], *Sm* in Tomato (*S. lycopersicum*) [23], *Yr5/Yr15* in Wheat (*Triticum aestivum*) [24], and *OsBRW1* in Rice (*Oryza sativa*) [25], demonstrate the power of R-gene–mediated resistance.

Sweetpotato (*Ipomoea Batatas* (L.) Lam.) is an allohexaploid (2n = 6x = 90) characterized by extreme heterozygosity, self- and cross-incompatibility, and a complex subgenome structure [26]. These attributes render classical map-based cloning exceptionally challenging [27]. The genetic complexity of polyploids has historically hindered the dissection of resistance traits. However, our team has recently established a robust foundation for genetic analysis in sweetpotato. In our latest study published in *Nature Plants* [28], we constructed a genome-wide allele dosage variation map based on deep sequencing of 294 hexaploid accessions. This work not only identified dosage quantitative trait loci (QTLs) for 23 agronomic traits but also demonstrated that breeding has progressively increased the dosage of favorable alleles to enhance trait performance. Furthermore, we validated the critical role of allele dosage in regulating gene expression and phenotypic variation through transgenic approaches. Despite these advances in genomic resources and the establishment of dosage-aware GWAS methodologies, the specific genetic architecture of resistance to BSRR remains uncharacterized. There is a critical gap in linking the identified dosage variations to the molecular mechanisms governing sweetpotato immunity against this pathogen.

Genome-wide association studies (GWAS) circumvent the requirement for biparental populations and provide high-resolution mapping of trait-associated variants [29], making them particularly suitable for dissecting polygenic resistance. GWAS has led to major advances in diploid crops such as rice [30,31,32], wheat [33,34,35], and maize [36,37,38], enabling the discovery of causal genes and informing marker-assisted selection [39]. In polyploids, however, methodological challenges, including accurate allele dosage calling, repetitive genome regions, and incomplete reference assemblies, have hampered progress [40,41,42]. The recent release of high-quality genomes for the sweetpotato cultivars ‘Beauregard’ [43] and ‘Xushu18’ [44], along with the diploid progenitor *Ipomoea trifida* [45,46,47], now provides a robust foundation for GWAS in this crop [28,48,49,50,51]. Yet, no study has systematically mapped BSRR resistance loci across the sweetpotato genome or integrated genetic associations with transcriptional responses to infection.

Here, we present the first GWAS of BSRR in hexaploid sweetpotato. Previous studies have established ‘Guangshu87’ (GS87) and ‘Xinxiang’ (XX) as widely recognized resistant and susceptible reference cultivars [20], respectively. This study integrates two years of field phenotyping across 135 core accessions with SNP genotyping, and is complemented by multi-timepoint RNA-seq analysis utilizing publicly available transcriptomic data for these two accessions with extreme phenotypes under pathogen challenge. We identified nine QTLs exhibiting consistent allelic dosage effects, pinpointed candidate genes within significant QTL intervals, and distinguished between constitutive and infection-induced regulators. Collectively, our work not only identifies significant SNP loci and candidate genes for breeding BSRR-resistant varieties but also establishes a framework for dissecting complex disease resistance in polyploid crops lacking haplotype-resolved genomes.

## 2. Materials and Methods

### 2.1. Plant Materials

A panel of 294 sweetpotato core accessions was provided by the Sweetpotato Research Group, Institute of Crops, Guangdong Academy of Agricultural Sciences. After excluding accessions with missing samples or incomplete phenotypic records, 135 accessions were retained for subsequent analyses (Table S1). Whole-genome resequencing data have been deposited in the National Center for Biotechnology Information (NCBI) under BioProject accession number PRJNA1089346. Field trials were conducted in August 2024 and July 2025 at the National Sweetpotato Germplasm Nursery in Guangzhou, China (23°39′ N, 113°44′ E). A randomized complete block design (RCBD) was implemented, with three replicates per accession. Each replicate consisted of five plants grown in a single row. Standard agronomic practices were followed, with row spacing of 110 cm, within-row spacing of 20 cm, and ridge height of 30 cm.

### 2.2. Disease Resistance Evaluation

The bacterial strain used for inoculation was *D. dadantii* Ech36, maintained in our laboratory. The strain was first activated on nutrient agar plates (5.0 g/L tryptone, 3.0 g/L beef extract, 1.0 g/L yeast extract, 10.0 g/L sucrose, and 15.0 g/L agar powder; pH 7.2) at 30 °C and subsequently transferred to nutrient broth liquid medium (same composition as above but without agar) for shaking culture at 30 °C and 200 rpm until reaching the logarithmic growth phase. The resulting bacterial suspension was adjusted to a final concentration of 1 × 10^7^ CFU/mL. Inoculation was performed three times: (i) prior to transplanting, seedling bases were immersed in the bacterial suspension for 20 min; (ii) and (iii) stem spray inoculations were performed twice at 7-day intervals thereafter, targeting only the stem apex to avoid contact with leaves. Seven days after the final inoculation, resistance was assessed based on the extent of stem lesion development (Table S2).

**Figure 1:**
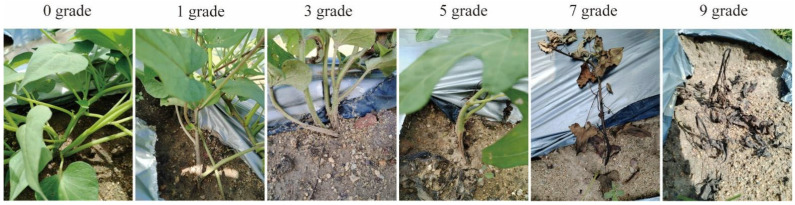
Six-class disease rating scale for BSRR resistance in *I. batatas* seedlings. Disease severity ranged from 0 (no symptoms) to 9 (severe wilting, necrosis, or plant death).

Because BSRR typically manifests as systemic vascular browning and stem maceration in field conditions—and no high-throughput automated phenotyping method is currently available—disease resistance for all 135 accessions was assessed visually. We adapted an indoor screening protocol described previously [14] and refined it according to observed field disease progression to establish a six-class disease rating scale suitable for field assessment. To ensure data reliability, the experiment was arranged in three independent field blocks (biological replicates) per year, with each block comprising five individual plants per accession. Furthermore, disease grades for each plant were determined through consensus evaluation by three independent experts to minimize subjective bias. Disease ratings were assigned based on symptom severity as follows: 0 grade: No visible symptoms, normal plant growth. 1 grade: Slight stem browning, no wilting. 3 grade: Localized brown lesions, not girdling the stem. 5 grade: Lesions girdling the stem with longitudinal extension, stems prone to breakage at the inoculation site. 7 grade: Black-brown maceration at the inoculation site with extensive lesion spread, and obvious whole-plant wilting. 9 grade: Plant death (Figure 1).

Based on the disease ratings, the Disease index (DI) for each accession was calculated using the following formula: DI= ∑(Number of plants at each disease rating×corresponding disease rating)/(highest disease rating×total number of plants)×100. Accessions were subsequently categorized into five resistance classes based on DI: Highly resistant (HR), DI ≤ 20; Resistant (R), 20 < DI ≤ 40; Moderately resistant (MR), 40 < DI ≤ 60; Susceptible (S), 60 < DI ≤ 80; Highly susceptible (HS), 80 < DI ≤ 100.

Broad-sense heritability (*H*^2^) was estimated from DI values obtained in two environments using a linear mixed model, in which environment was treated as a fixed effect and genotype, genotype-by-environment interaction, and residual error were treated as random effects. Variance components were estimated via restricted maximum likelihood (REML). The formula for broad-sense heritability was calculated as: $H^{2}=\frac{\sigma_{G}^{2}}{\sigma_{G}^{2}\;+\frac{\sigma_{GE}^{2}}{e}\;+\frac{\sigma_{e}^{2}}{e\cdot r}}$, where $\sigma_{G}^{2}$ is the genotypic variance; $\sigma_{GE}^{2}$ is the genotype-by-environment interaction variance; $\sigma_{e}^{2}$ is the residual variance; *e* = 2 environments; *r* = 3 replicates per environment. Analyses were conducted using the lme4 package (v1.1-35.5) in R (v4.5.2).

To account for environmental variation and isolate the underlying genetic effects, phenotypic values were adjusted using best linear unbiased prediction (BLUP). BLUPs were estimated using lme4 (v1.1-35.5) with the linear mixed model: Phenotype ~ (1|Year) + (1|Line) + (1|Year: Line) + (1|Year: Replicate). Pairwise comparisons of the trait values were performed using Student’s *t*-test with the ‘stat_compare_means’ function in ggpubr (v.0.6.0).

### 2.3. Resequencing Data and SNP Calling

SNP genotyping data were obtained from our previously published dataset [28]. A total of 6,828,068 high-quality SNPs and 1,679,029 insertion–deletion variants were used in the present study.

### 2.4. Genome-Wide Association Analysis

Given that sweetpotato is an allohexaploid, haplotype-based analyses were not applicable. In the GWAS, the BLUP values of the DI were used as the phenotypic parameter to measure BSRR resistance. GWAS was conducted using SNP dosage data filtered by minor allele frequency (≥0.1) and missing rate (≤0.5), employing a linear mixed model that accounted for population structure and relative kinship, as implemented in the R package GWASpoly (v.2.10) [41]. First, the set. K function was used to compute the covariance matrix for the polygenic background effect. Subsequently, the GWASpoly function was used to perform GWAS using an ‘additive’ model, where the SNP effect was proportional to minor allele dosage. Significance thresholds were determined by permutation testing (1000 permutations; α = 0.05), yielding a genome-wide cutoff of *p* < 10^−5^ (−log_10_(*p*) > 5). Significant associations were identified by grouping adjacent significant SNPs (within 500 kb) exhibiting a pairwise linkage disequilibrium (LD) *r*^2^ ≥ 0.5, as calculated using the ldseq package (v.2.1.5) [52] in R (v4.3.2). The resulting genomic intervals were further assessed, and those containing fewer than 2 SNPs with *p* < 10^−5^ or fewer than 10 SNPs with *p* < 10^−3^ were filtered out. Candidate genes were defined as those located within each associated interval and within 50 kb upstream or downstream.

Using the *I. trifida* reference genome, all genes located within the significant QTL regions identified through GWAS were extracted to form the candidate gene set. Functional annotation of all candidate genes was performed using the InterProScan online platform (https://www.ebi.ac.uk/interpro/ (accessed on 1 February 2026)) to identify conserved protein domains. Genes were subsequently classified into functional categories based on the presence of domain architectures commonly associated with plant disease resistance. These categories included: F-box/LRR (COI1-like): genes containing both an F-box domain and a leucine-rich repeat (LRR) domain, homologous to the *Arabidopsis* COI1 protein; U-box E3 ligases: genes harboring a U-box domain, involved in the ubiquitin–proteasome pathway; Kinase-only (non-RLK): genes encoding proteins with a kinase domain but lacking transmembrane regions and extracellular LRR motifs, distinguishing them from canonical receptor-like kinases (RLKs); bHLH/NAC transcription factor: genes containing either a bHLH or an NAC DNA-binding domain; Other: genes not fitting into the above categories.

### 2.5. PCA of SNP Dosage Within Significant QTL Regions

To assess whether accessions with contrasting disease resistance levels could be distinguished based on genetic variation within significant QTL regions, principal component analysis (PCA) was performed using SNP dosage data extracted from all significant QTL intervals identified in the GWAS. The input matrix included only SNPs located within these defined QTL regions (as described in Section 2.4). Dosage values ranged from 0 to 6, corresponding to the number of alternative alleles present at each locus in the allohexaploid sweetpotato genome. PCA was conducted in R (v4.3.2) using the ‘prcomp’ function. Accessions were color-coded according to their disease resistance classifications: Group II (R), Group III (MR), Group IV (S), and Group V (HS). The first two principal components (PC1 and PC2) were visualized with 95% confidence ellipses for each group, and the proportion of variance explained by each component was indicated on the axis labels. Figures were generated using the ‘ggplot2’ package (v3.4.0).

### 2.6. Identification of Differentially Expressed Genes (DEGs) in Response to D. dadantii Infection

Transcriptome datasets for the moderately resistant cultivar ‘GS87’ and the highly susceptible cultivar ‘XX’ at 0 h (DEG_T0), 18 h (DEG_T18), and 30 h (DEG_T30) post-inoculation with *D. dadantii* were retrieved from the NCBI Sequence Read Archive (BioProject PRJNA1162560). Raw RNA-seq reads were quality-filtered using Trimmomatic (v0.93) [53]. Cleaned reads were then aligned to the *I. trifida* reference genome using STAR (v2.7.7a) [54]. Gene-level raw read counts for protein-coding genes were generated from the resulting BAM alignment files using featureCounts (v1.6.3) [55] and normalized to Fragments Per Kilobase of transcript per Million mapped fragments (FPKM). DEGs between the moderately resistant ‘GS87’ and the susceptible ‘XX’ in response to *D. dadantii* infection were identified using DESeq2 (v1.36.0) [56], applying false discovery rate (FDR) < 0.05 and an absolute |log_2_(fold change)| ≥ 1 as significance thresholds.

### 2.7. RNA Isolation and Quantitative Real-Time PCR Validation

#### 2.7.1. Sample Preparation

To validate the transcriptomic profiles obtained from the publicly available RNA-seq data, an independent biological experiment was conducted following the identical experimental workflow. Stem cuttings (25 cm in length, each bearing six leaves) were excised from healthy sweetpotato plants and pre-cultured in sterile water for 4 d to promote adventitious root formation and growth, following the protocol of Xie et al. [20]. The basal ends of the cuttings were freshly wounded (without damaging the newly formed roots) and immediately immersed in 50 mL of *D. dadantii* suspension (1 × 10^7^ CFU/mL) for 30 min. After inoculation, cuttings were thoroughly rinsed with sterile water. All inoculated plants were maintained in sterile water under controlled environment conditions: 30 °C, 90% relative humidity, and a 13 h light/11 h dark photoperiod. Basal stem segments (1 cm in length) were harvested at 0, 18, and 30 h post-inoculation, immediately flash-frozen in liquid nitrogen, and stored at −80 °C. Each treatment included three biological replicates.

#### 2.7.2. qRT-PCR Validation

Total RNA was extracted from 1 cm basal stem segments and used for qRT-PCR. First-strand complementary DNA (cDNA) was synthesized from 500 ng of total RNA using the FastKing gDNA Dispelling RT SuperMix kit (Tiangen Biotech, Beijing, China). The resulting cDNA was diluted 10-fold prior to qRT-PCR analysis. Gene-specific primers were designed using Primer3Plus (Table S3). qRT-PCR reactions were performed on a Bio-Rad CFX96 Real-Time PCR Detection System (Bio-Rad, Hercules, CA, USA) using the SYBR Green Pro Taq HS Premix qPCR Kit (RuiZhen Biotechnology Co., Ltd., Guangzhou, China) in a final reaction volume of 10 μL. Cycling conditions were initial denaturation at 95 °C for 3 min, followed by 40 cycles of 95 °C for 5 s, 60 °C for 30 s, and 65 °C for 5 s, with a final extension at 95 °C for 5 min. Each sample included three biological replicates and three technical replicates. *α-Tubulin* served as the internal reference gene. Relative gene expression was calculated using the 2^−ΔΔCt^ method, comparing expression across 0, 18, and 30 hpi between the resistant (‘GS87’) and susceptible (‘XX’) cultivars. Linear regression models were used to analyze temporal expression dynamics in GraphPad Prism 10 (v.10.1.2; https://www.graphpad.com/).

### 2.8. Statistical Analysis

Phenotypic data were processed and statistically analyzed using Microsoft Excel 2021 and SPSS v20.0 (International Business Machines Corporation, Armonk, NY, USA). All results are presented as the mean of three independent measurements. One-way analysis of variance (ANOVA) was conducted to evaluate experimental error, and Pearson correlation coefficients among phenotypic traits were calculated using SPSS v20.0.

## 3. Results

### 3.1. Phenotypic Diversity and Resistance to BSRR in Sweetpotato

A total of 135 sweetpotato core accessions were evaluated for resistance to bacterial stem rot under controlled artificial inoculation in two independent field environments: August 2024 (Aug_2024) and July 2025 (Jul_2025). Disease severity was assessed on individual plants using a standardized six-class rating scale (Figure 1). Plants displaying atypical symptoms attributable to non-pathogenic factors (e.g., insect damage or mechanical injury) were excluded from analysis. For all valid plants, the DI was calculated using a weighted scoring formula, yielding values from 0 to 100 per replicate. The phenotypic value for each accession in a given year was defined as the arithmetic mean of DI across three biological replicates. BLUP values derived from the combined two-year, three-replicate dataset were used as the input phenotype for GWAS (Table S4).

In Aug_2024, DI values ranged from 31.11 to 100.00 (mean = 78.83; coefficient of variation (CV) = 17.17%). In Jul_2025, the DI distribution was broader, with values ranging from 18.52 to 100.00 (mean = 75.27; CV = 22.6%), indicating greater phenotypic dispersion in this environment (Table 1). A weak but significant positive correlation was observed between years (*r* = 0.23, *p* < 0.01; Figure S1), suggesting partial cross-environment consistency but overall low phenotypic stability. The broad-sense *H*^2^ estimated from the two-year, three-replicate dataset was 0.35, indicating moderate genetic control of DI but substantial influence from environmental variation and genotype-by-environment interaction (G × E).

**Figure 2:**
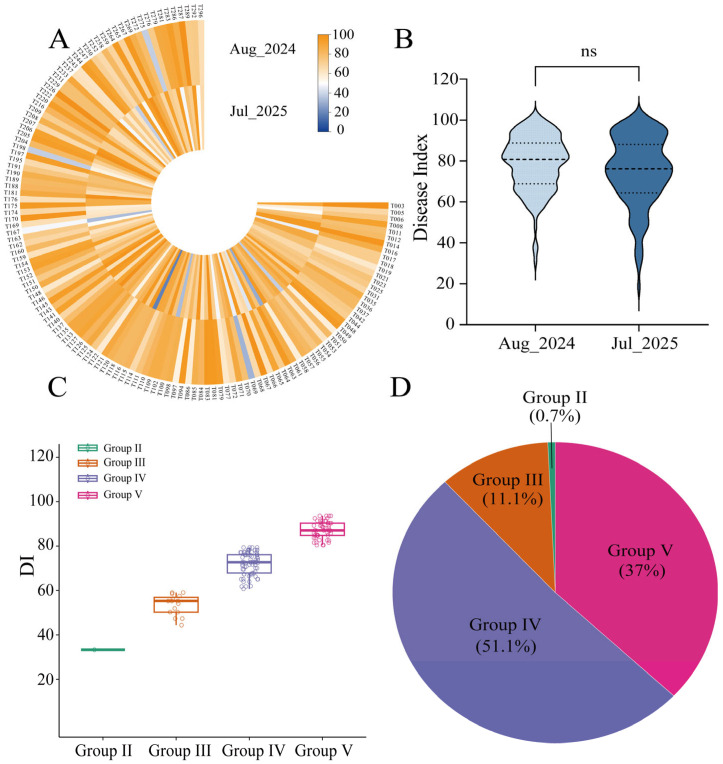
Phenotypic diversity and classification of BSRR resistance (expressed as DI) in sweetpotato core germplasm. (**A**) Repeatability of DI across two environments. Orange: susceptible; blue: resistant; intensity indicates severity of response. (**B**) Violin plots comparing DI distributions across environments. ns indicates no significant difference (*p* > 0.05). (**C**) Distribution of 135 sweetpotato accessions across four resistance groups based on BLUP values. (**D**) Proportion of accessions in each group.

A heatmap of BLUP-based DI values revealed substantial phenotypic divergence among the 135 accessions (Figure 2A). Violin plots showed approximately symmetric, unimodal DI distributions in both environments, with density peaks near the means (Figure 2B), consistent with a quantitative trait architecture, suggesting that resistance to bacterial stem rot in sweetpotato is likely polygenic. Based on BLUP values, the 135 accessions were classified into four resistance categories: Group II (R; *n* = 1, 0.7%); Group III (MR; *n* = 15, 11.1%); Group IV (S; *n* = 69, 51.1%); and Group V (HS; *n* = 50, 37.0%) (Figure 2C,D; Table S5).

### 3.2. GWAS Identifies QTLs Associated with BSRR Resistance in Sweetpotato

**Figure 3:**
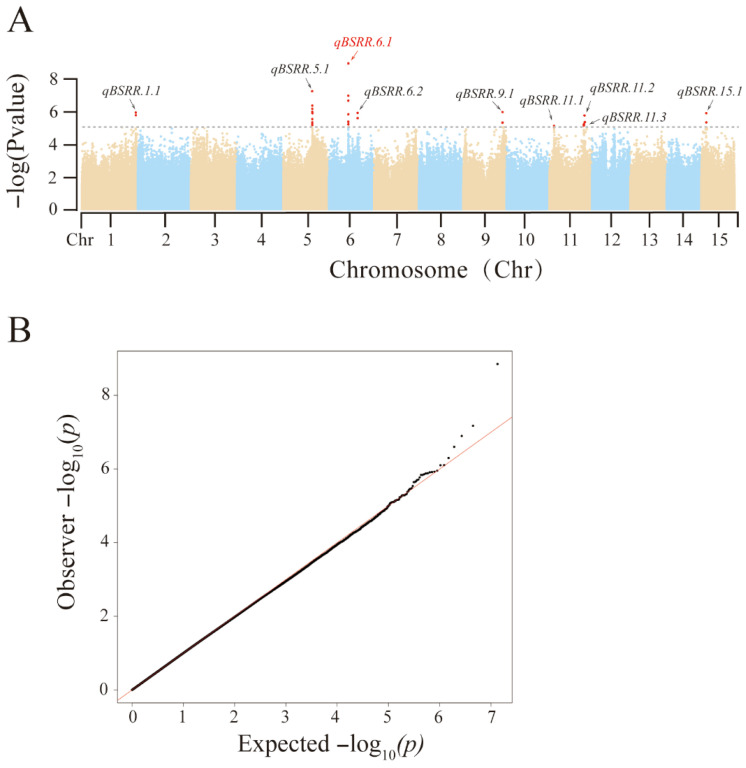
GWAS for BSRR resistance in hexaploid sweetpotato. (**A**) Manhattan plot displaying −log_10_(*p*-value) for SNP associations with the BLUP values of the DI across the genome. (**B**) Quantile–quantile (Q–Q) plot comparing observed and expected −log_10_(*p*-values). The red line represents the expected distribution, the dashed lines indicate the 95% confidence interval, and the black dots represent the observed SNPs. The plot indicates well-controlled population structure and minimal inflation of test statistics.

To elucidate the genetic determinants of BSRR resistance in sweetpotato, 135 core accessions were evaluated under artificial inoculation in two independent field environments (Aug_2024 and Jul_2025). BLUP was employed to account for environmental variation and produce reliable phenotypic values for subsequent analysis. Following stringent quality control (minor allele frequency ≥ 0.1; missing rate ≤ 0.5), a total of 6,828,068 high-quality SNP markers were retained for GWAS. Using the R package GWASpoly (v2.1.0), a linear mixed model was fitted, and a significance threshold of −log_10_(*p*) > 5 was applied. Nine significant QTLs were identified, distributed across six linkage groups (Chr1, Chr5, Chr6, Chr9, Chr11, and Chr15), and designated as qBSRR.1.1, qBSRR.5.1, qBSRR.6.1, qBSRR.6.2, qBSRR.9.1, qBSRR.11.1, qBSRR.11.2, qBSRR.11.3, and qBSRR.15.1 (Figure 3). These QTL regions collectively harbored 23 significant SNPs and encompassed 156 candidate genes (Table S6). Among these loci, the major-effect QTL qBSRR.6.1 (located at Chr6: 13,420,528 bp) exhibited the strongest association signal (−log_10_(*p*) = 8.85) and comprised 11 candidate genes (Table S6). Allelic dosage analysis showed that individuals homozygous for the T allele across all six SNP sites (T/T/T/T/T/T) displayed the highest BLUP values (i.e., lowest resistance), whereas the balanced heterozygous genotype (T/T/T/C/C/C; 3T:3C) exhibited intermediate resistance. Notably, the C-biased genotype (T/C/C/C/C/C; 1T:5C) did not confer strong resistance, suggesting that resistance at qBSRR.6.1 is not solely governed by C/T allele dosage but likely depends on specific dosage ratios or allelic combinations (Figure S2).

In addition to the major locus, we prioritized two candidate genes, *IbTCP5* and *IbERF003*, located within the flanking QTL intervals qBSRR.5.1 and qBSRR.6.2, respectively, for preliminary functional assessment. These genes were selected based on their physical position and functional annotations as putative susceptibility factors. Both genes exhibited significantly higher expression levels in the susceptible cultivar ‘XX’ compared to the moderately resistant cultivar ‘GS87’ at 0 h post-inoculation (Figure S3). Although the specific genotypes at these loci were not determined for the two cultivars, this expression pattern is consistent with a potential role in promoting susceptibility: the elevated basal levels in the susceptible ‘XX’ likely predispose the host to infection, whereas the suppressed expression in the resistant ‘GS87’ may contribute to its resistance phenotype.

To further evaluate the putative biological functions of the 156 candidate genes, we performed domain annotation using InterProScan. Most genes (112) were classified as “other/unknown function,” including proteases, RNA-binding proteins, metabolic enzymes, and proteins containing domains of unknown function. The remaining genes included six non-receptor kinases (“kinase-only”), three U-box E3 ubiquitin ligases, two F-box/LRR proteins, one bHLH transcription factor, and one NAC transcription factor. Notably, we did not identify any canonical NLR R-genes containing the characteristic NB-ARC domain among these candidates (Figure S4; Table S7).

### 3.3. Significant QTL SNPs Effectively Distinguish Resistance Categories

**Figure 4:**
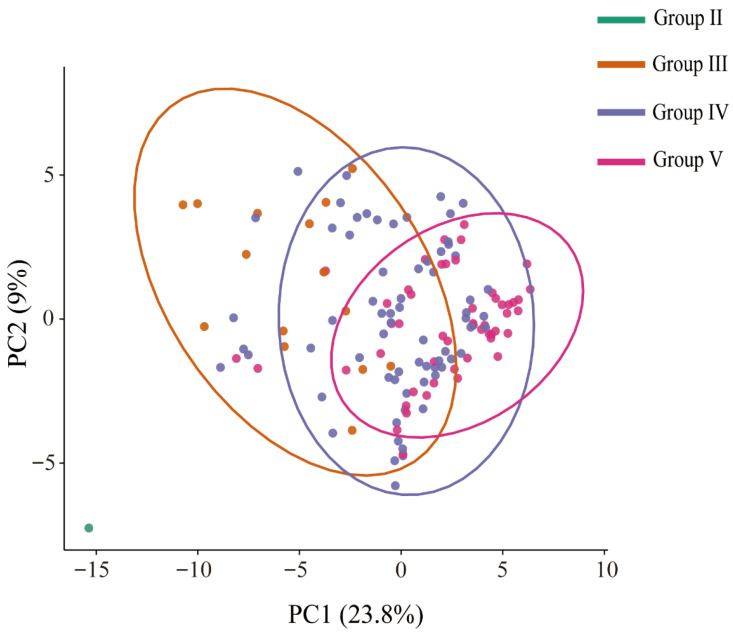
PCA based on significant SNPs within the nine BSRR-associated QTL regions. Each dot represents one accession, colored according to DI category: Group II (R, green), Group III (MR, orange), Group IV (S, blue), and Group V (HS, pink). Solid lines in the legend correspond to the group colors. Ellipses indicate 95% confidence intervals. The single resistant accession (T115) is clearly separated from all other groups along PC1, indicating a distinct genetic background.

To evaluate the discriminatory power of the identified QTL regions, PCA was performed using SNP dosage data extracted exclusively from the significant loci. The first principal component (PC1) accounted for 23.8% of the total genetic variation and revealed a distinct stratification pattern: resistant accessions were predominantly distributed at the negative end, whereas susceptible accessions clustered at the positive end, with intermediates occupying intermediate positions (Figure 4). While this distribution indicates that the SNP combinations within these QTLs effectively capture the major resistance-associated genetic structure, we acknowledge that complete separation of accessions was not achieved. This overlap is likely attributable to the inherent genetic complexity of the trait. Specifically, factors such as the current sample size, genetic variation not fully captured by the marker set, and other unknown biological variables may collectively contribute to this residual confounding. Nevertheless, these SNPs represent strong candidates for developing molecular markers to facilitate the rapid screening and selection of BSRR-resistant germplasm.

### 3.4. Integration of GWAS and RNA-Seq Identifies Candidate Genes Associated with BSRR Resistance

**Figure 5:**
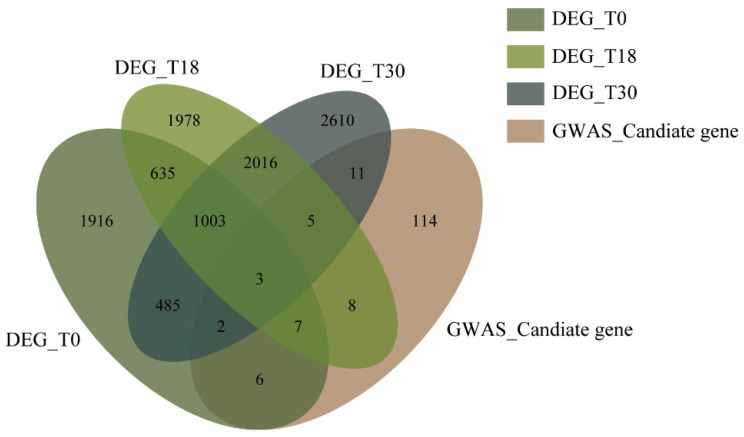
Venn diagram illustrating overlap between DEGs at T0, T18, and T30 and GWAS candidate genes.

To identify high-confidence candidate genes associated with BSRR response, we integrated GWAS and RNA-Seq results. Differential expression analysis of RNA-Seq data identified 4057, 5655, and 6135 DEGs at 0, 18, and 30 h post-inoculation, respectively. Intersecting these DEGs with the 156 GWAS-derived candidate genes yielded 62 high-confidence candidates (Figure 5; Table S8). Expression pattern analysis revealed distinct temporal dynamics for these overlapping genes (Table 2). Notably, the majority of these 62 overlapping genes exhibited consistently higher expression in the susceptible cultivar ‘XX’ than in the moderately resistant ‘GS87’ across later time points, suggesting that a broad transcriptional reprogramming favoring susceptibility may occur in compatible interactions. However, amidst this predominant susceptibility-associated trend, we discerned a distinct subset of candidates exhibiting divergent expression dynamics. Specifically, we prioritized four representative genes—*IbPUB4*, *IbKCS5*, *IbLig1*, and *IbPUB25*—that embody two contrasting regulatory strategies: *IbPUB4*, *IbKCS5*, and *IbLig1* were markedly upregulated or induced in the resistant cultivar ‘GS87’, suggesting they may act as positive regulators of immunity; conversely, *IbPUB25* maintained constitutively elevated expression in the susceptible cultivar ‘XX’, pointing to its potential function as a susceptibility factor. Spatial mapping of these candidates onto the Venn diagram further validated their specific temporal signatures: *IbPUB4* localized to the T0/T30 intersection, *IbKCS5* to the T0-specific cluster, *IbLig1* to the T0/T18 overlap, and *IbPUB25* to the constitutive intersection spanning all time points (T0, T18, and T30).

### 3.5. qRT-PCR Validation of BSRR Candidate Gene Expression

**Figure 6:**
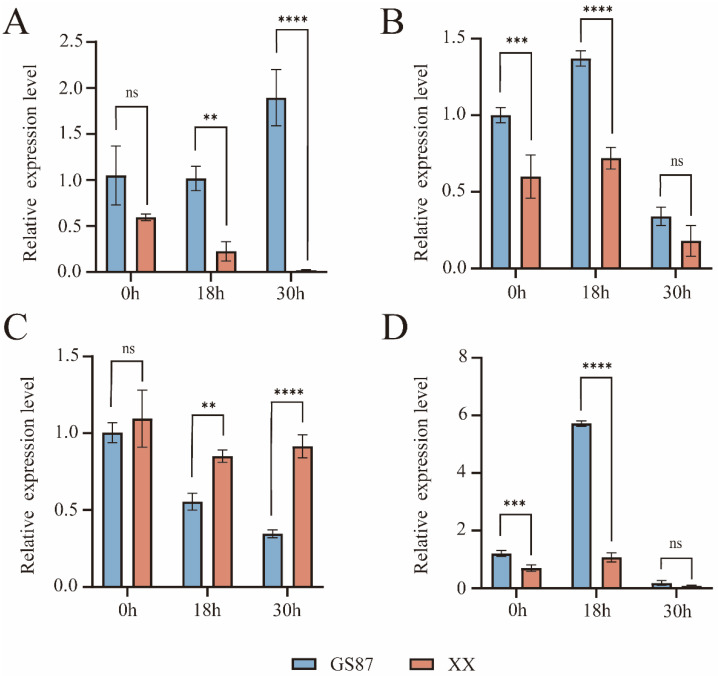
Relative expression levels of four candidate genes in sweetpotato cultivars ‘GS87’ and ‘XX’ at 0, 18, and 30 h after inoculation. (**A**) *IbPUB4*. (**B**) *IbKCS5*. (**C**) *IbPUB25*. (**D**) *IbLig1*. Data represent means ± SD (*n* = 3 biological replicates). Statistical significance between cultivars at each time point was assessed using a two-tailed Student’s *t*-test. Significance levels are indicated as: **, *p* < 0.01; ***, *p* < 0.001; ****, *p* < 0.0001; ns, not significant.

To validate the expression patterns of the resistance-associated candidates identified from the RNA-Seq analysis (as described in Section 3.4), we performed qRT-PCR to assess the expression levels of the four prioritized candidates (*IbPUB4*, *IbKCS5*, *IbPUB25*, and *IbLig1*) in stem tissues of ‘GS87’ and ‘XX’ following inoculation with *D. dadantii*. Relative expression at each time point was calculated using the mean expression of both cultivars as the calibrator. Overall, qRT-PCR results closely mirrored RNA-Seq expression trends, reinforcing the reliability of the transcriptome dataset and supporting the potential involvement of these genes in BSRR response (Tables S9–S14, where detailed FPKM values and statistical analyses are provided). Four genes displayed distinct temporal and cultivar-specific expression patterns following inoculation with *D. dadantii*. *IbPUB4* expression increased progressively in ‘GS87’, reaching its highest level at 30 h, whereas it declined steadily in ‘XX’ over the same period (Figure 6A). *KCS5* showed consistently higher expression in ‘GS87’ than in ‘XX’ at all time points, peaking at 18 h and slightly decreasing at 30 h (Figure 6B). In contrast, *PUB25* expression remained relatively stable in ‘XX’ but decreased gradually in ‘GS87’, resulting in a greater divergence between the two cultivars by 30 h (Figure 6C). *Lig1* was induced strongly in ‘GS87’ at 18 h, while its expression remained low throughout the time course in ‘XX’ (Figure 6D). These patterns suggest that the differential regulation of these genes may contribute to cultivar-specific responses to BSRR.

## 4. Discussion

The broad-sense heritability (*H*^2^ = 0.35) estimated for BSRR in this study suggests a quantitative genetic basis, differing from the high heritability typically associated with qualitative traits controlled by major-effect genes. This implies that BSRR in sweetpotato is polygenic and sensitive to environmental factors. Such a pattern is consistent with the genetic complexity of auto-allohexaploid species, where high heterozygosity and subgenome redundancy often obscure the effects of individual loci, thereby complicating the identification of single causal genes. Despite these challenges, the observed heritability supports the application of GWAS, particularly when enhanced by multi-environment phenotyping. Although our association panel was moderate in size (*n* = 135), its extensive genetic diversity, combined with high-depth resequencing (average coverage ≥ 20×) and a polyploid-aware analytical pipeline (GWASpoly), provided sufficient statistical power to detect major associations despite the genetic complexity. Previous studies in sweetpotato have successfully used similar panel sizes to dissect resistance to weevil [48], root-knot nematodes [57], and Fusarium root rot [58], validating this approach for complex traits in polyploids. Notably, the reliability of the identified loci is further supported by the convergence of multi-omics data. The significant GWAS peaks show spatial congruence with candidate genes (*IbPUB4*, *IbKCS5*, and *IbLig1*) that exhibit pronounced and biologically consistent differential expression patterns. This independent transcriptomic evidence offers strong support for the potential involvement of these candidates in resistance mechanisms, thereby helping to mitigate concerns regarding statistical power limitations inherent to moderate-sized panels in polyploid crops.

Phenotypic evaluation across two field seasons revealed a scarcity of strong resistance to *D. dadantii* within the tested germplasm. Only one accession was classified as resistant and 15 as moderately resistant, while the majority (119 accessions) were susceptible. This distribution likely reflects both the high virulence of the pathogen and the limited availability of resistant sources in current breeding pools. Additionally, disease severity was higher in August 2024 than in July 2025. This variation corroborates reports that *D. dadantii* virulence increases with temperature [4], highlighting the influence of environmental conditions on BSRR. Population structure analysis indicated that the resistant accession (T115) was genetically distinct from the susceptible groups. Statistical analysis of the BLUP values further confirmed T115 as a significant outlier; its value (33.28) was 3.49 standard deviations lower than the population mean (75.41 ± 12.08). This distinct positioning suggests that its resistance likely stems from a unique allelic combination. The phenotypic overlap observed among the intermediate groups further corroborates the quantitative nature of the observed partial resistance. Given that the identified QTLs generally show small-to-moderate effects, the rarity of highly resistant accessions suggests that favorable alleles at multiple loci are rarely combined in existing germplasm. This highlights the necessity of marker-assisted pyramiding to accumulate these alleles in future breeding programs.

Our GWAS identified nine QTLs associated with BSRR resistance. The major locus, qBSRR.6.1, exhibited a distinct dosage effect: accessions with a balanced heterozygous genotype (T/T/T/C/C/C) showed higher resistance compared to those with C-biased genotypes. While this pattern suggests a potential role for allele dosage or non-additive interactions, validation in segregating populations is required to confirm the specific genetic mode of action. This dosage-dependent resistance underscores the unique genetic architecture of allohexaploid sweetpotato. Genomic mapping reveals that key candidates within this region, such as *IbPUB4*, exist as functional homologs distributed across distinct linkage groups. This structural complexity likely provides the genetic basis for the allele dosage effects observed in our model.

The qBSRR.6.1 interval contains 11 candidate genes, 9 of which have functional annotations. Notably, no canonical plant immune receptors, such as NLRs, RLKs, or WRKY transcription factors, were identified in this region. Instead, the candidates are annotated with functions related to transcriptional regulation, protein complex assembly, metabolism, and signal transduction. The absence of classical R-genes in this major QTL suggests that resistance to *D. dadantii* in sweetpotato may involve quantitative regulatory mechanisms rather than a single major resistance gene, a scenario also observed in other polyploid crops like wheat and potato. Integrating GWAS signals with transcriptome data at 0, 18, and 30 h post-inoculation identified four high-confidence candidate genes. *IbPUB4*, *IbKCS5*, and *IbLig1* appeared to be positive regulators, showing induced expression in the resistant cultivar. In contrast, *IbPUB25* seemed to act as a susceptibility factor, with higher expression in the susceptible cultivar. qRT-PCR validation confirmed these patterns. These results suggest a dual strategy for breeding: silencing *IbPUB25* to remove negative constraints, and pyramiding favorable alleles of *IbPUB4*, *IbKCS5*, and *IbLig1* to boost defense. However, as these functions are inferred from expression and homology, future reverse-genetic studies are needed for confirmation.

*IbPUB4*, annotated as a U-box domain-containing protein, belongs to a family of E3 ubiquitin ligases widely implicated in plant stress responses and adaptation [59,60]. PUB proteins mediate substrate ubiquitination for 26S proteasome-dependent degradation to fine-tune immune signaling [61,62,63]. In ‘GS87’, *IbPUB4* expression increased during infection, while it decreased in ‘XX’. This suggests that *IbPUB4* could potentially enhance resistance by relieving immune suppression, similar to *VyPUB21* in grapevine [64]. Thus, *IbPUB4* is proposed as a promising marker for resistance screening.

*IbKCS5* encodes a key enzyme in very-long-chain fatty acid (VLCFA) biosynthesis [65]. VLCFAs are precursors for cuticular waxes and suberin, which form barriers against pathogens [66]. In *Arabidopsis*, KCS5 and KCS6 both contribute to wax production [67,68]. The specific induction of *IbKCS5* in ‘GS87’ points to a potential role in strengthening epidermal barriers to limit *D. dadantii* spread, making it a target for breeding varieties with better cuticle integrity.

*IbPUB25* showed consistently higher expression in the susceptible cultivar, suggesting a negative role in resistance. While the sweetpotato homolog *IbPUB52* is a negative regulator under drought [69], *Arabidopsis AtPUB25/26* prevents excessive immune activation by ubiquitinating BIK1 [70,71,72]. Therefore, high *IbPUB25* expression in susceptible lines may dampen defense signaling, implying that alleles with lower expression could be beneficial for breeding. Furthermore, *IbTCP5* and *IbERF003*, located in neighboring QTLs (qBSRR.5.1 and qBSRR.6.2), showed higher basal expression in the susceptible cultivar ‘XX’ than in the resistant ‘GS87’, suggesting they might act as negative regulators.

*IbLig1* encodes DNA ligase, essential for DNA repair and genome stability [73,74,75]. Its stronger induction in ‘GS87’ suggests a role in repairing pathogen-induced DNA damage, highlighting an underexplored aspect of sweetpotato immunity that may be related to genome maintenance.

**Figure 7:**
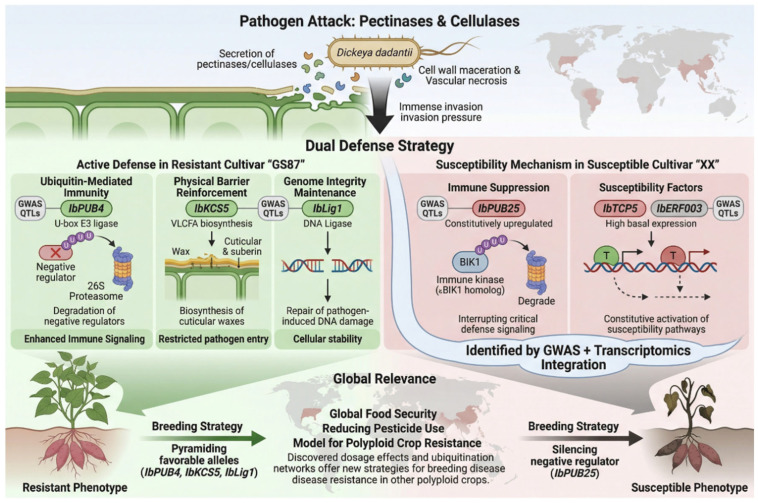
A model of the “Dual Defense Strategy” in hexaploid sweetpotato against *D. dadantii*.

Collectively, identifying *IbPUBs*, *IbKCS5*, and *IbLig1* as key candidates—against the notable scarcity of canonical NLR R-genes in sweetpotato [76,77]—suggests that this species relies on a multi-layered quantitative strategy. Accordingly, such a mechanism likely favors durable immune homeostasis, offering a distinct advantage over the unstable R-gene arms races observed in other pathosystems. Building on this, the proposed ‘dual-strategy’—silencing susceptibility factors (e.g., *IbPUB25*) while pyramiding positive regulators (e.g., *IbPUB4*, *IbKCS5*)—provides a strategic blueprint for breeding other polyploid root and tuber crops like potato [78]. Given their shared challenges of genomic complexity and soil-borne pathogens [79], these species could potentially leverage such markers to mitigate the persistent yield-resistance trade-off. Amid climate change and expanding pathogen ranges [80], deploying these evolutionarily conserved modules for signal modulation and barrier reinforcement represents a promising avenue for enhancing global food security (Figure 7).

In summary, this study identified nine QTLs and several candidate genes associated with BSRR resistance in sweetpotato using an integrated GWAS and transcriptomics approach. The results suggest that resistance is quantitative and may involve mechanisms related to ubiquitin-mediated regulation, cell wall reinforcement, and genome maintenance. While the hexaploid nature of sweetpotato presents significant challenges for immediate functional validation of these candidates, the identified QTLs and expression markers provide valuable resources for marker-assisted selection. Future efforts should focus on validating the specific functions of these candidates and pyramiding their favorable alleles to accelerate the development of resistant varieties.

## 5. Conclusions

In this study, integrating multi-environment GWAS with transcriptomics identified nine QTLs for BSRR resistance, highlighting the major locus qBSRR.6.1 with a distinct dosage effect. We prioritized four key candidates: *IbPUB4*, *IbKCS5*, and *IbLig1* may function as positive regulators of immunity—likely enhancing defense via ubiquitin-mediated signaling, cuticular wax biosynthesis, and DNA repair, respectively. Conversely, *IbPUB25* is suggested to act as a susceptibility factor, potentially suppressing immune responses. This functional divergence underscores a dual breeding strategy: silencing *IbPUB25* to remove negative constraints while pyramiding favorable alleles of *IbPUB4*, *IbKCS5*, and *IbLig1* to bolster active defense. Additionally, *IbTCP5* and *IbERF003*, located in adjacent QTLs, exhibited higher basal expression in the susceptible cultivar, suggesting background-specific roles in resistance regulation.

## Figures and Tables

**Table 1:** Summary statistics for DI in sweetpotato core germplasm across two field environments.

Environment	Min	Max	Mean	Skewness	Kurtosis	CV (%)
Aug_2024	31.11	100.00	78.83	−0.77	0.81	17.17%
Jul_2025	18.52	100.00	75.27	−0.75	0.42	22.6%

**Table 2:** Intersection analysis of GWAS candidate genes with DEGs across time points.

Time Point	Total Overlap	Upregulated Genes	Downregulated Genes
GWAS + DEG_T0	18	9	9
GWAS + DEG_T18	23	18	5
GWAS + DEG_T30	21	18	3

## Data Availability

All data supporting the findings of this study are available within the article and its Supplementary Materials. Further inquiries should be addressed to the corresponding author.

## Supplementary Materials

The following supporting information can be downloaded at: https://www.mdpi.com/article/10.3390/biology15080643/s1. Figure S1: Phenotypic distribution of disease index (DI) values and cross-environment correlation among sweetpotato core accessions; Figure S2: Allelic dosage effect of the major QTL qBSRR.6.1 on bacterial stem and root rot severity in sweetpotato; Figure S3. Relative expression levels of (A) *IbTCP5* and (B) *IbERF003* in sweetpotato cultivars ‘GS87’ and ‘XX’ at 0, 18, and 30 h post-*D. dadantii* inoculation, as quantified by qRT-PCR; Figure S4. Functional classification of candidate genes located within the qBSRR.6.1 genomic interval in sweetpotato; Table S1. Summary information of 135 sequenced sweetpotato accessions; Table S2. Phenotypic data of disease grade for the evaluated sweetpotato germplasm; Table S3. Primers used in this study; Table S4. DI values of selected sweetpotato accessions evaluated across two environments and their corresponding BLUP estimates; Table S5. Distribution of 135 sweetpotato accessions across four resistance groups based on BLUP values; Table S6. List of candidate genes located within all QTLs associated with BSRR resistance in sweetpotato; Table S7. Candidate genes identified within all significant QTL intervals, classified according to conserved protein domains and functional categories; Table S8. Candidate genes co-localized between GWAS signals and DEG analyses under BSRR stress, including physical positions and functional annotations; Table S9. DEGs between CK-GS87 and CK-XX groups; Table S10. DEGs between T0-GS87 and T0-XX groups; Table S11. DEGs between T18-GS87 and T18-XX groups; Table S12. DEGs between T30-GS87 and T30-XX groups; Table S13. Raw read counts for all genes across all samples. These data serve as the input for differential expression analysis; Table S14. Normalized gene expression levels (FPKM) for all samples.
