# Supplementary material for: Genome-Wide Association Study and Candidate Gene Identification for Resistance to Bacterial Stem and Root Rot in Sweetpotato
Source: Biology (Basel). 2026 Apr 19;15(8):643. doi: 10.3390/biology15080643 (PMC13113871; doi:10.3390/biology15080643)
Supplement: Supplementary file 1 [file biology-15-00643-s001.zip › Supplementary Figure.pdf]

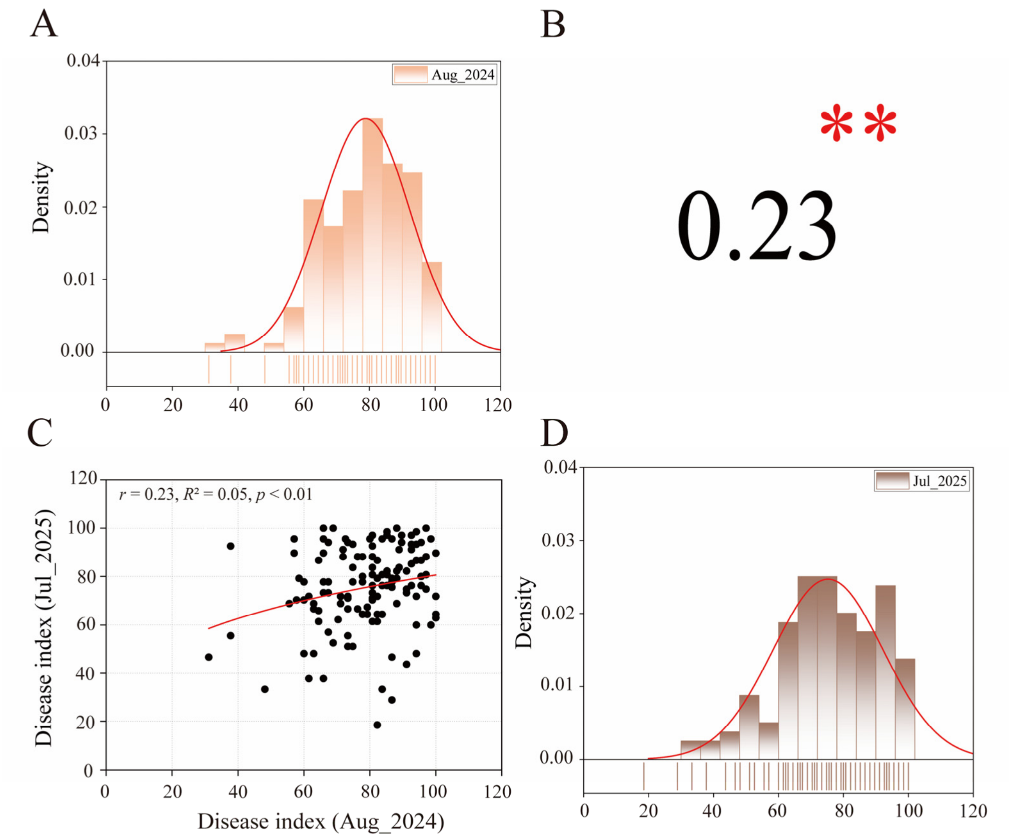

Supplementary Figure S1. Phenotypic distribution of disease index (DI) values and cross-environment correlation among sweetpotato core accessions.

(A–D) Phenotypic analysis of DI in 135 sweet potato accessions evaluated across two field environments. **(A)** Density histogram of DI in Aug_2024, with tick marks at the bottom indicating individual data points; **(B)** Pearson correlation coefficient ($r = 0.23$, $p < 0.01$) between DI values in the two environments; **(C)** Scatter plot showing the relationship between DI in August 2024 and July 2025 ($r = 0.23$, $R^2 = 0.05$, $p < 0.01$); **(D)** Density histogram of DI in July 2025, with tick marks indicating individual accession values. ** indicates significance at $p < 0.01$.

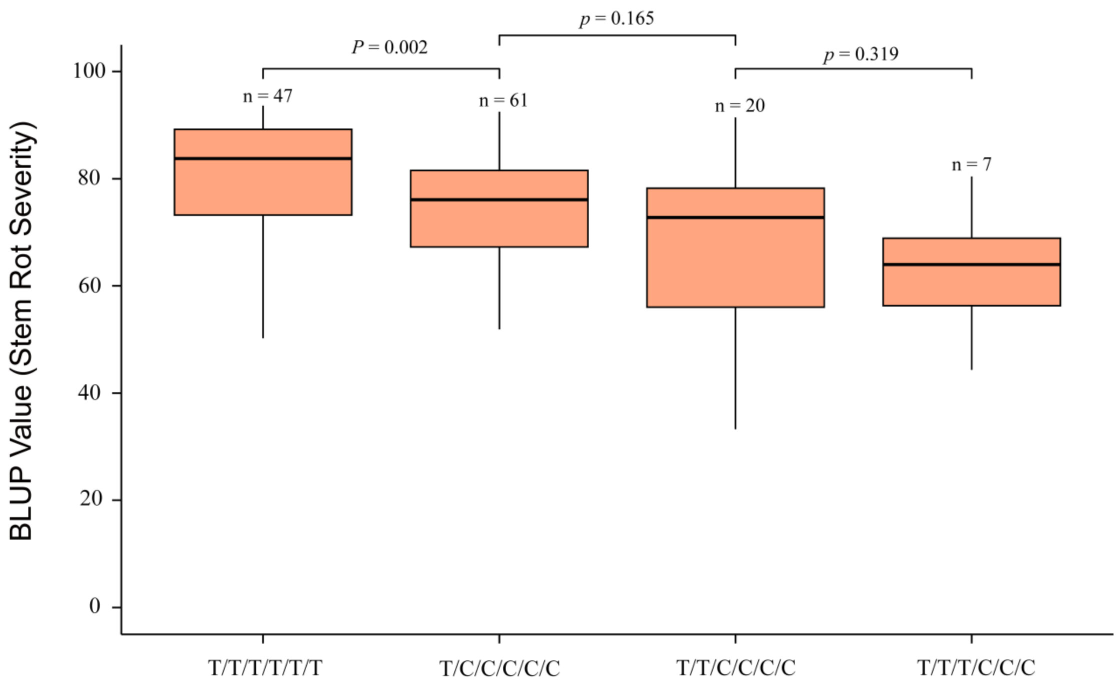

Supplementary Figure S2. Allelic dosage effect of the major QTL qBSRR.6.1 on bacterial stem and root rot severity in sweetpotato. The x-axis represents the hexaploid genotype dosage, where T denotes the reference allele and C denotes the alternative allele. Boxplots show the BLUP values (higher values indicate more severe disease) for each dosage class. Boxes represent the interquartile range (IQR, 25th–75th percentiles) with the horizontal line indicating the median, and whiskers extending to 1.5× the IQR. Pairwise Wilcoxon rank-sum tests were performed between groups, with *P*-values shown above the comparisons.

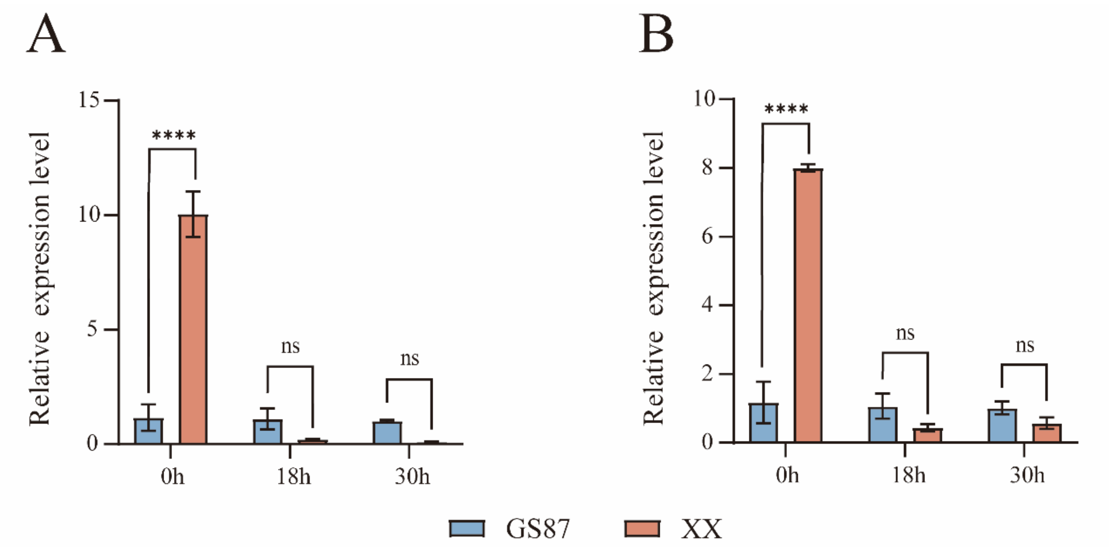

Supplementary Figure S3. Relative expression levels of (A) *IbTCP5* and (B) *IbERF003* in sweetpotato cultivars ‘GS87’ and ‘XX’ at 0, 18, and 30 h post-*D. dadantii* inoculation, as quantified by qRT-PCR. Data represent means ± SD (n = 3 biological replicates). Statistical significance between cultivars at each time point was assessed using a two-tailed Student's *t*-test. Significance levels are indicated as: ****, $P < 0.0001$; ns, not significant.

A

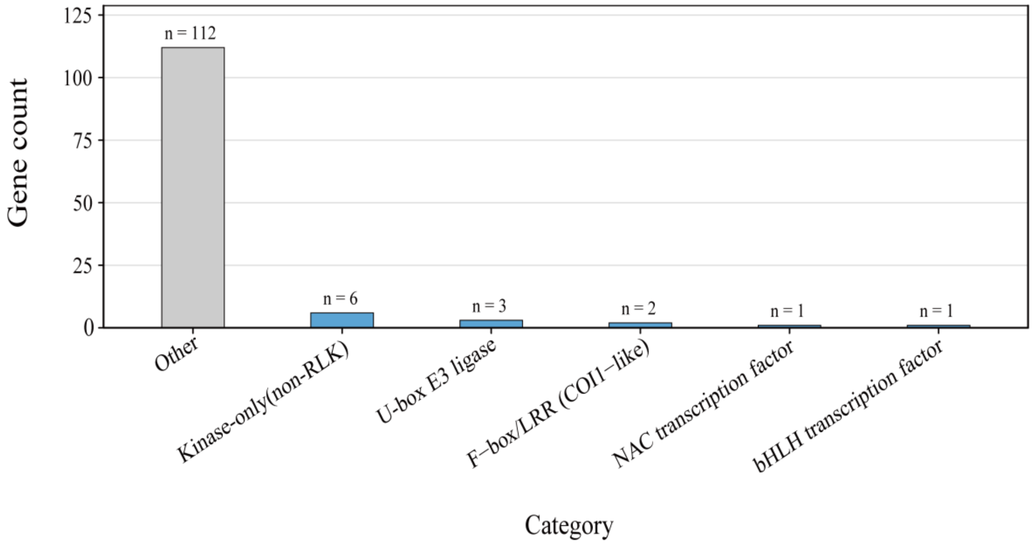

B

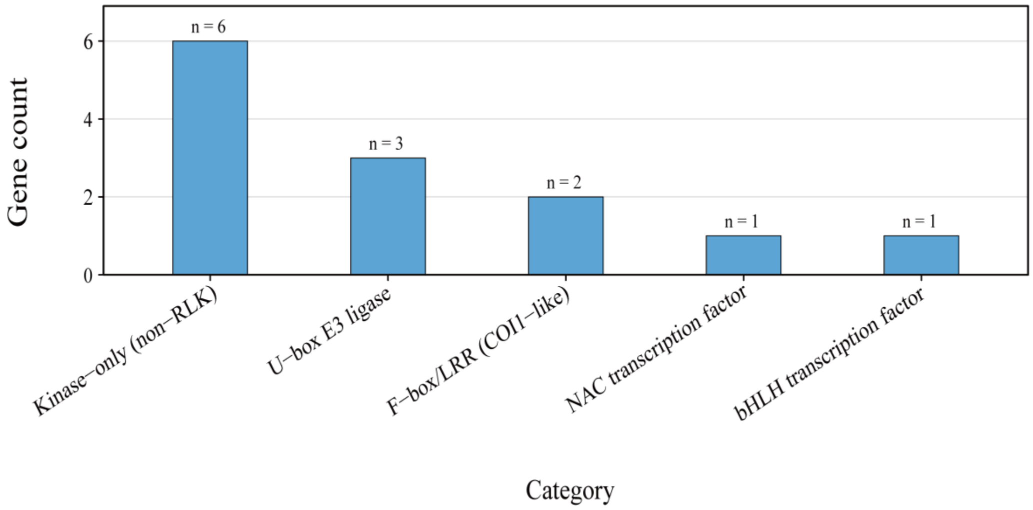

Supplementary Figure S4. Functional classification of candidate genes located within the qBSRR.6.1 genomic interval in sweetpotato. **(A)** Distribution of all candidate genes across functional categories. The "Other" category includes proteins with domains such as proteases, RNA-binding proteins, metabolic enzymes, and Domains of Unknown Function (DUF). **(B)** Expanded view of non-"Other" candidates, highlighting defense-related gene families.
